# Level of Adequate Knowledge of Non-communicable Diseases and Associated Factors Among Adult Residents of North Shewa Zone, Oromia Region, Ethiopia: A Mixed-Method Approach

**DOI:** 10.3389/fpubh.2022.892108

**Published:** 2022-06-23

**Authors:** Elsabeth Legesse, Tadesse Nigussie, Derara Girma, Leta Adugna Geleta, Hiwot Dejene, Berhanu Senbeta Deriba, Tinsae Abeya Geleta, Degemu Sahlu, Mengistu Tesema, Ayele Tilahun, Mukemil Awol, Firanbon Teshome, Gachana Midaksa, Feyiso Bati

**Affiliations:** ^1^Department of Public Health, College of Health Science, Salale University, Fitche, Ethiopia; ^2^Department of Nursing, College of Health Science, Salale University, Fitche, Ethiopia; ^3^Department of Midwifery, College of Health Science, Salale University, Fitche, Ethiopia; ^4^Department of Health Behavior and Society, Faculty of Public Health, Institute of Health, Jimma University, Oromia, Ethiopia; ^5^Department of Public Health, College of Health Sciences, School of Public Health, Mizan Tepi University, Mizan Tepi, Ethiopia; ^6^Department of Public Health, College of Health Sciences, Dire Dawa University, Dire Dawa, Ethiopia

**Keywords:** non-communicable disease, knowledge, associated factors, adult, Ethiopia

## Abstract

**Background:**

Non-communicable diseases (NCDs) are currently the leading cause of morbidity and mortality, posing significant challenges to global healthcare systems. Particularly, the prevalence of NCDs is rising in Ethiopia, resulting in a triple burden of diseases on the health system that disproportionately affects all age groups. Hence, this study aims to determine the level of adequate knowledge of NCDs and associated factors among adult residents of the North Shewa zone, Oromia region, Ethiopia.

**Methods:**

A community-based cross-sectional study with a concurrent mixed-method approach was conducted from April 1, 2021 to May 30, 2021 among 846 residents using the multistage sampling technique. Interviewer administered questionnaire was used to collect quantitative data and a guiding checklist was used to collect qualitative data. Bivariable and multivariable logistic regressions were fitted to compute the association between explanatory variables and knowledge of NCDs. Adjusted odds ratios at 95% confidence interval with a *p*-value < 0.05 were used to decree statistical significance in multivariable analysis. Also, a thematic framework analysis was used for qualitative data analysis.

**Results:**

A total of 823 subjects have participated in this study making a response rate of 97.3%. The level of adequate knowledge was 33.9% (95%CI: 30.67, 37.13). Higher-income, receiving information from health professionals, owning a TV, having a family member with NCD(s), and marital status were factors significantly associated with adequate knowledge of NCDs.

**Conclusion:**

This study reveals a high level of inadequate knowledge of NCDs despite its foundational ability in tackling the burden of NCDs. As a result, broadening a wider and more comprehensive health promotion strategy for the prevention of triple burden of NCDs would benefit the population. Additionally, special efforts are needed both at the practice and policy levels targeting the disadvantaged groups, such as low-income people, those who do not receive information from health professionals, those who do not own a television, and those who are widowed/divorced, who were found to have less knowledge of NCDs.

## Introduction

Non-communicable diseases (NCDs) are a group of conditions that include cardiovascular diseases, chronic respiratory diseases, cancers, and diabetes (1). However, the term “NCDs” has come to refer to a wide range of health concerns, including hepatic, renal, and gastroenterological diseases, endocrine, hematological, and neurological disorders, dermatological conditions, genetic disorders, trauma, mental disorders, and disabilities (e.g., blindness and deafness) (2).

According to the World Health Organization (WHO) (2021), NCDs kill 41 million people each year, accounting for 71% of all deaths worldwide, with 77% of NCD deaths occurring in low- and middle-income countries (LMICs). Subsequently, the most common NCD is cardiovascular disease, which kills 17.9 million people each year, followed by cancer (9.3 million), respiratory disorders (4.1 million), and diabetes (1.5 million) (3). This unnoticed epidemic is a major cause of poverty and a significant impediment to many countries' economic growth (4).

Tobacco use, physical inactivity, harmful alcohol use, and unhealthy diets are all linked to an increased risk of dying from NCDs (3). Furthermore, modifiable risk factors like high blood pressure, obesity, elevated blood sugar level, and high blood cholesterol, as well as non-modifiable risk factors like age, gender, genetic factors, race, and ethnicity, were revealed to increase the risk of NCDs (5–7). Nonetheless, detection, screening, and treatment of NCDs, as well as palliative care, are all important parts of the NCD response (3, 8).

Many NCDs have historically been linked to economic growth and termed “diseases of the rich.” However, nowadays, the burden of NCDs has increased in developing countries (7), while a dramatic decrease in NCDs-related burden is recorded in developed countries (9). As a result of aging, increased unplanned urbanization, and globalization of unhealthy lifestyles, the burden of NCDs and associated risk factors continues to rise in African countries (10).

Concerning knowledge of NCDs, studies conducted among diverse populations in different countries have revealed varying levels of knowledge of NCDs. Accordingly, a good level of knowledge was reported to be 81.2% in Malaysia (11), 57.9% in Bangladesh (12), 46.7% in Spain (13), 43.8% in Saudi Arabia (14), 43% in Sri Lanka (15), 27.7% in Malaysia (16), 25% in China (17), and 12.5% in Myanmar (18). Furthermore, a finding from China indicates chronic diseases knowledge varied from 29.5 to 90.2% (19).

Regarding factors associated with knowledge of NCDs; being females (12, 19), age (11, 12), higher level of education (16, 20, 21), higher wealth index (18), higher income (12), having health insurance (19), having a family history of chronic disease(s) (17–19), participating in society discussion (19), and more frequently gathering with friends/relatives (19), not smoking (12, 15), physical activity (12), receiving NCDs health information and self-care instructions from their physicians (17) were found to heighten the likelihood of having an adequate level of knowledge about NCDs.

In a particular, the prevalence of NCDs is increasing in Ethiopia, resulting in a triple burden of diseases to the health system (i.e., communicable diseases, NCDs, and injuries) that disproportionately affects all age groups (22). Subsequently, the likelihood of dying prematurely from one of the main NCDs is 17.4% for males and 16.9% for females in Ethiopia (23). Furthermore, the country's NCD mortality is primarily attributed to cardiovascular diseases (CVDs) and cancer (24). Concerningly, NCDs have been identified as future threats that Ethiopia's health sector will be unable to address on its own, necessitating multisectoral collaboration (25). Predominantly, inadequate knowledge of NCDs is one of the principal causes of high mortality, along with inadequate screening, early detection, and treatment, and insufficient diagnostic and treatment facilities available in the country (22).

Overall, the epidemic of NCDs poses challenging health consequences for individuals, families, and communities, and threatens to overwhelm healthcare systems (3). Moreover, knowledge gaps about NCDs and their risk factors in the general population are significant barriers to effective NCD prevention and treatment (26). However, despite the enormous challenge that NCDs pose, the level of adequate knowledge and associated factors remain unidentified in Ethiopia (22, 27). As a result, a need for further study on the knowledge of NCDs is suggested (28). Hence, the present study aims to determine the level of adequate knowledge of NCDs and associated factors among adult residents of the North Shewa zone, Oromia region, Ethiopia. Given all of the research gaps and needs for further investigation in Ethiopia, conducting such a study will result in early detection, a reduction in the NCD burden, and guidance in the development of an effective policy.

## Methods and Materials

### Study Area, Design, and Period

The study was conducted in the North Shewa zone, Oromia region, Ethiopia. The zone has a total area of 10,322.48 km^2^ and a population density of 138.66 people per km^2^, with 13 rural districts and two town administrations. Furthermore, the Zone has a total population of approximately 1,639,586 people, of whom 717,552 are men and reside in 521,506 households. Fiche town – the zone's capital – is 112 kilometers north of Addis Ababa –Ethiopia's capital. Additionally, the zone has 64 health centers and five public hospitals that provide health care services to the community. A community-based cross-sectional study design with a concurrent mixed-method approach was conducted from April 1, 2021 to May 30, 2021.

### Populations and Eligibility Criteria

A source population consisted of all adults over the age of 18 who were permanent residents (individuals who live at least 6 months) of a selected town in the North Shewa zone. A study population was randomly selected adult residents for a quantitative study. A qualitative study was conducted among purposely selected residents, health care providers, and public health experts.

### Sample Size, Sampling Frame, and Participants Recruitment

The sample size was determined using a single population proportion formula in Epi Info STAT CALC. version 7.2 based on the assumptions of a 95% confidence level, 5% margin of error (d), and a 50% proportion (p) of knowledge of NCDs. Because no similar study had previously been conducted in Ethiopia, 50% was taken. After applying a design effect of 2 and a 10% non-response rate, the final sample size obtained was 846. Additionally, a total of five sessions of Focus Group Discussions (FGDs) were conducted among residents in the selected districts. Each FGD was composed of 10 residents. Additionally, 10 In-Depth Interview (IDI) was conducted among health care providers with different professionals and public health experts. Regarding a sampling technique, a multistage sampling technique was used. Towns in the zone were purposely selected since the prevalence of NCDs is high among urban populations. Thirty percent of the towns in the zone were randomly selected by lottery method, namely Fiche, Kuyu, Debretsige, Mukaturi, and Gundomeskel town. Consequently, 30% of their kebeles (small administration units) were included in the study. The sample size was proportionally allocated to each town. Finally, a computer-generated simple random sampling technique was used to select households from the kebeles using house numbers. In a household with more than one adult individual, the lottery method was used to select one of them. For the qualitative part, the purposive sampling technique was employed considering maximum variability with the assumption of obtaining relevant data to supplement the research objectives. The intention was to explore detailed information on NCDs knowledge and healthy lifestyle in society.

### Data Collection Tools, Personnel, and Procedures

The data collection tool was developed after reviewing previously done studies and it has four sections including; socio-demographic, exposure to NCDs information, knowledge of NCDs, and practices of healthy lifestyles (15, 29–32). The knowledge measuring tool was reliable in this study (Cronbach-alpha = 0.72). Ten experienced BSc nurses were recruited for data collection, and onsite supervision was provided by five BSc in public health supervisors to facilitate data collection procedures on a daily basis. Data were collected through a face-to-face interview by using a pre-tested questionnaire. Additionally, FGDs and IDIs were used to get participants' experiences related to knowledge of NCDs. By using an open-ended guiding list of questions, five sessions of FGDs were undertaken to explore the knowledge of NCDs and the healthy life practice of the community. The FGDs were moderated by an experienced health professional and note taker. During the discussion, notes were taken and their voices were recorded using a tape recorder. Similarly, 10 sessions of IDIs were conducted among health care providers and public health experts.

### Study Variables

The dependent variable of the study was the level of adequate knowledge of NCDs. Knowledge was assessed by 32 questions related to respondents' knowledge about NCDs and their risk factors. Correct answers were given a score of 1 and incorrect answers were given 0. The total possible score ranged from 0 to 32. A cut-off level ≥60%, of the individual percentage scores, was selected as an indicator of adequate knowledge (15). As well, in the current study the tool has an acceptable reliability (Cronbach-alpha = 0.81). The explanatory variables were socio-demographic (age, sex, religion, marital status, educational status, occupational status, income, and ethnicity), exposure information about NCDs (getting information from media, getting information from health professionals, getting information from family members, having family members with NCDs and having friends with NCDs) and behavioral factors (physical activity, alcohol and tobacco use).

### Data Processing and Analysis

The collected data were checked for completeness manually and entered, cleaned, and checked by Epi data manager version 4.0.2 and then exported to SPSS version 23 for analysis. Descriptive analysis of different variables was done based on their nature. Bivariable analysis was done using binary logistic regression for all independent variables and a *p*-value < 0.25 was used to consider candidate variables for the multivariable analysis. Finally, a multivariable binary logistic regression analysis was done to control for possible confounders and to identify factors significantly associated with an adequate level of knowledge of NCDs at a *p*-value of < 0.05 with a 95% confidence interval adjusted odds ratio. Model fitness was checked using the Hosmer and Lemeshow goodness of fit model and it was fitted (*p*-value = 0.12). as well, the variance inflation factor was set at five to detect multicollinearity among explanatory variables, and no multicollinearity was found. Besides, before analyzing data, all FGDs and IDIs were transcribed in Afan Oromo text by replaying the recorded voice from tape and the notes taken during discussion. Then, the Afan Oromo version text was translated into English language. Different ideas in the text were merged in their thematic areas and thematic framework analysis was done manually. The results were presented in narration by triangulating with quantitative findings.

### Ethical Consideration

Ethical approval was obtained from the ethical review committee of Salale University. A permission letter was obtained from the respective district and kebele administration before data collection. Written informed consent was obtained from each study participant.

## Results

### Socio-Demographic Characteristics

A total of 823 respondents have completed the study making a response rate of 97.3%. The mean age (standard deviation) of the respondents was 31.83 (11.04) years. Of the total participants, more than half, 443 (53.8%) were females. Regarding marital status, 497 (60.4%) of them were married. Approximately, one-third, 255 (31%) of them attended above secondary education. Furthermore, the majority, 628 (76.3%) of the participants were from the Oromo ethnic group. Additionally, nearly a fourth, 189 (23%) of them were a merchant (Table 1).

**Table 1:** Socio-demographic characteristics of adult residents of selected towns in North Shewa zones, Oromia region, central Ethiopia, 2021.

**Variables**	**Categories**	**Frequency (*n* = 823)**	**Percentage**
Age group	18–24	230	27.9
	25–29	205	24.9
	30–34	113	13.7
	>34	275	33.4
Sex	Male	380	46.2
	Female	443	53.8
Marital status	Single	270	32.8
	Married	497	60.4
	Divorced	24	2.9
	Widowed	32	3.9
Religion	Orthodox	607	73.7
	Protestant	176	21.5
	Muslim	24	2.9
	Others	16	1.9
Educational status	No education	153	18.6
	Primary	212	25.8
	Secondary	203	24.7
	Above secondary	255	31.0
Occupation	Housewife	162	19.7
	Gov't employee	187	22.7
	Farmer	91	11.1
	Merchant	189	23.0
	Students	154	18.7
	Others	40	4.9
Ethnic background	Oromo	628	76.3
	Amhara	160	19.4
	others	35	4.3
Monthly income	= <3,578	341	56.6
	>3,578	261	43.4

### Knowledge of NCDs

**Figure 1:**
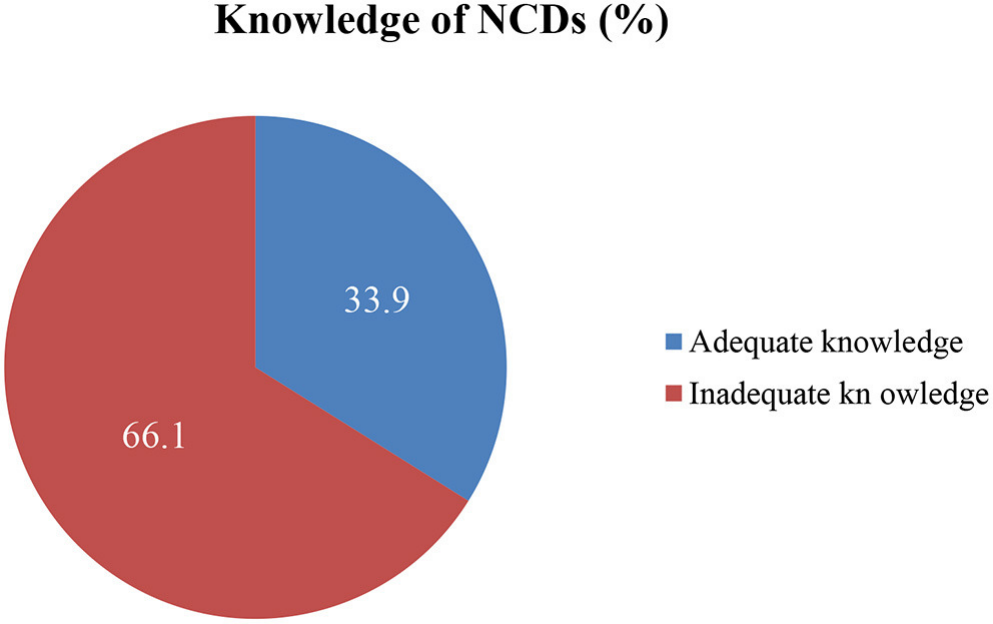
Knowledge level of NCDs among adult residents of selected towns of north Shewa zone, Oromia region, central Ethiopia, 2021.

On the 32 items tool measuring respondents' knowledge about NCDs, the mean score (standard deviation) of respondents was 14.6 (4.34). Of the total, 279 (33.9%) of the participants had an adequate level of knowledge (Figure 1). Regarding the causes of NCDs, more than two-thirds, 568 (69.0%) stated that NCDs are caused by supernatural power (God). In line with this, a misconception about the cause of the disease was observed among FGD participants. For instance, a 31 years old male discussant said “… *Eating vegetables can lead to chronic diseases such as diabetes and hypertension due to their low nutritional value and weakness in increasing protection against these diseases*…” Regarding the onset time, about 446 (54.2%) of the study participants said that NCDs onset only at an older age (Table 2). Similarly, participants of IDI explained that there is no sufficient knowledge in the community regarding NCDs. For example, a 34 years old female clinical nurse said that “*Because there is a lack of public awareness about these diseases, people seek medical attention after they develop complications*…” Also, a male public health expert of 29 years old working at a health center explained that the community has no awareness about NCDs. He has elucidated that “*In general, there is a lack of awareness among communities about their health status. They drink as much alcohol as they desire, and no one engages in physical activity... They are unconcerned about NCDs..*.”

**Table 2:** Knowledge measuring variables among adult residents of selected towns of north Shewa zone, Oromia region, central Ethiopia, 2021.

**Variable**	**Categories**	**Frequency (percentage) of a correct answer**
Perception of NCD	NCDs are caused by supernatural power	568 (69.0)
	NCDs occur only at old age	446 (54.2)
	NCDs are a public health problem	757 (92.0)
	NCDs cause premature death	696 (84.6)
Types of NCDs	Hypertension	752 (91.4)
	DM	682 (82.9)
	CHD	273 (33.2)
	COPD	86 (10.4)
	Cancer	309 (37.5)
	Accident	23 (2.8)
	Blindness	31 (3.8)
	Stroke	66 (8.0)
Risk factors of NCDs	Smoking	541 (65.7)
	Alcohol drinking	503 (61.1)
	Passive smoker	94 (11.4)
	Obesity	310 (37.7)
	Consuming junk food	281 (34.1)
	Stress	364 (44.2)
	Anxiety	151 (18.3)
	Consuming excess salt	409 (49.7)
	Lack of physical exercise	185 (22.5)
Complication of NCDs	Intracranial bleeding	166(20.2)
	Stroke	487 (59.2)
	Heart disease	112 (13.6)
	Premature death	696 (84.6)
Prevention method of NCDs	Quit smoking	449 (56.7)
	Avoid alcohol drinking	514 (64.9)
	Doing physical exercise	379 (47.9)
	Avoid excess salt	345 (43.6)
	Losing weight	243 (30.7)
	Eating healthy diet	365 (46.1)
	Timely screening	681 (83.3)

### Factors Associated With Knowledge of NCDs

In bivariable analysis, variables such as sex, age group, educational status, marital status, income, occupation, presence of TV, getting information from health professionals, having neighbors with NCDs, and presence of a family member(s) with NCDs were significantly associated with the knowledge of NCDs. In multivariable analysis, income, getting information from health professionals, owning a TV, having a family member with NCD, and being widowed/divorced were significantly associated with adequate knowledge of NCDs. Accordingly, respondents who earn a monthly income of ≥3,578 were almost two times more likely to have adequate knowledge about NCDs as compared with those who earn a monthly income of <3,578 (AOR = 1.68, 95% CI: 1.05–2.68). Respondents who received information from health professionals were more than twice more likely to have adequate knowledge of NCDs when compared with those do not receive information from health professionals (AOR = 2.29, 95% CI:1.53–3.45). Moreover, having family members with NCDs was associated with the knowledge of residents i.e., respondents who have family members with NCDs were more than two times more likely to have adequate knowledge of NCDs when compared to their counterparts (AOR = 2.23, 95% CI:1.49–3.35). Besides, respondents who possessed television were more than twice more likely to have adequate knowledge about NCDs as compared to those who don't possess television (AOR = 2.48, 95%CI:1.32–4.65). Additionally, the marital status of the respondents determines the level of NCDs knowledge. Thus, the likelihood of having adequate knowledge among divorced and widowed participants is 0.80 less likely as compared to unmarried participants (AOR = 0.20, 95%CI:0.07–0.59) (Table 3).

**Table 3:** Factors associated with knowledge of NCDs among adult residents of selected towns of north Shewa zone, Oromia region, central Ethiopia, 2021.

**Variables**	**Categories**	**Knowledge status**		**COR (95%CI)**	**AOR (95%CI)**
		**Adequate**	**Inadequate**		
Sex	Male	160	220	1.98 (1.48–2.65)	1.20 (0.76–1.89)
	Female	119	324	1	1
Marital status	Married	159	338	0.75 (0.55–1.02)	0.56 (0.31–1.01)
	Divorced & widowed	16	40	0.64 (0.34–1.19)	**0.20 (0.07**–**0.59)** ^*^
	Single	104	166	1	1
Educational status	Primary	163	49	0.69 (0.45–1.12)	0.62(0.33–1.17)
	Secondary	125	78	1.45 (0.93–2.26)	0.77 (0.40–1.48)
	Above secondary	149	106	1.66 (1.08–2.53)	1.27 (0.64–2.52)
	No education	107	46	1	1
Occupation	Gov't employee	82	105	2.41 (1.60–3.61)	0.96 (0.48–1.92)
	Merchant	71	118	1.85 (1.23–2.79)	1.24 (0.71–2.16)
	Students	59	95	1.91 (1.24–2.95)	1.07 (0.33–3.49)
	Students	5	35	0.44 (0.17–1.17)	0.37 (0.12–1.17)
	Housewife	62	191	1	1
Age group	>34	165	110	1.32 (0.92–1.91)	1.70 (0.86–3.37)
	30-34	72	41	1.13 (0.71–1.81)	1.11 (0.52–2.35)
	25-29	154	51	0.66 (0.43–1.00)	0.51 (0.25–1.04)
	<25	153	77	1	1
Income	>3578	144	117	2.27 (1.61–3.19)	**1.68 (1.05**–**2.68)** ^*^
	= <3578	251	90	1	1
Owning TV	Yes	400	232	1.78 (1.23–2.57)	**2.48 (1.32**–**4.65)** ^*^
	No	144	47	1	1
Getting information from HP	Yes	258	187	2.25 (1.67–3.05)	**2.13 (1.41**–**3.22)** ^*^
	No	286	92	1	1
Having family member with NCD	Yes	99	119	3.34 (2.42–4.61)	**2.21 (1.46**–**3.34)** ^*^
	No	445	160	1	1

*COR, Crude Odds Ratio; AOR, Adjusted Odds Ratio; CI, Confidence Interval; 1, References*;

* *significant at p-value < 0.05; HP, Health Professionals*.

### Exposure to Information About NCDs

**Figure 2:**
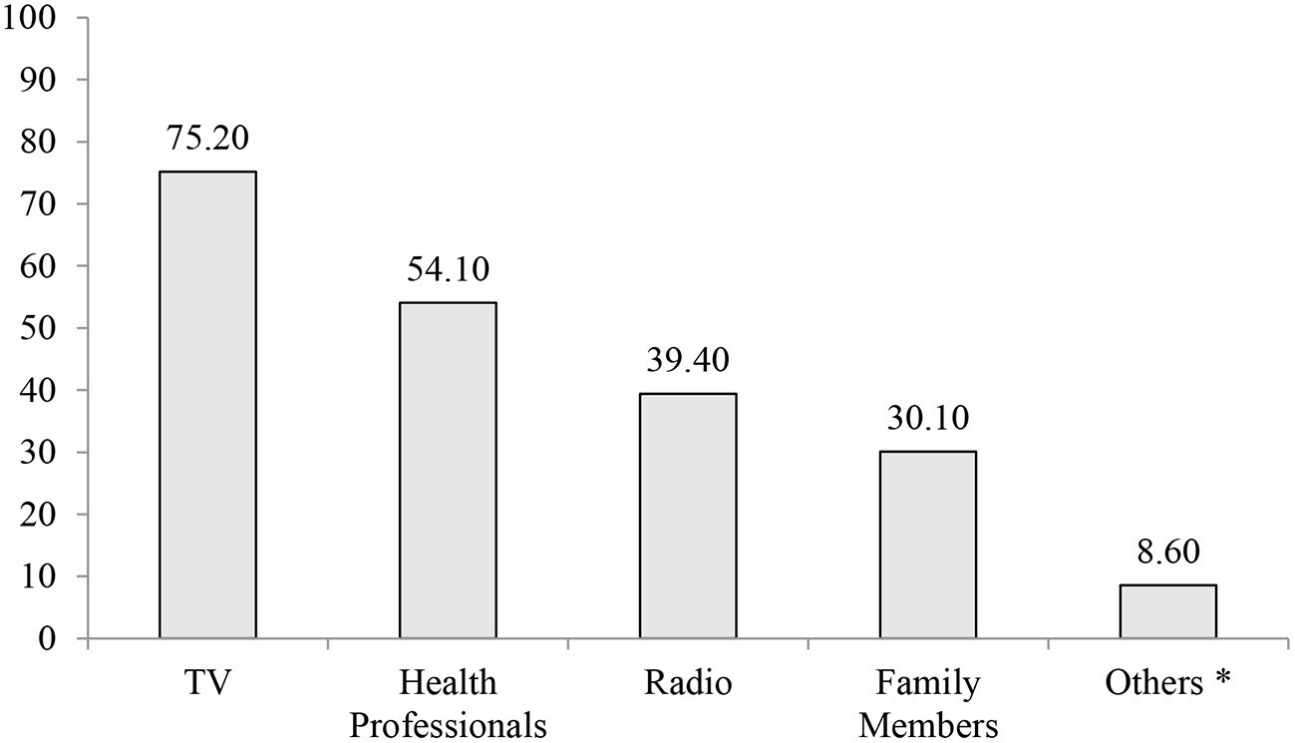
Sources of information about NCDs among adult residents of selected towns of north Shewa zone, Oromia region, central Ethiopia, 2021. *Friends, Neighbors, Newspapers.

Concerning information exposure, all of the study participants heard about NCDs. The majority of the participants, 619 (75.2%) of them heard about NCDs from Television (TV) (Figure 2). Comparable to this a 32 years old male participant in FGD stated that “*…I usually watch TV in my spare time, and there are advertisements on TV that promote healthy behavior such as reducing salt consumption, quitting smoking, and receiving immunization against diseases such as the human papillomavirus, which causes cervical cancer.…”*

Of the total, 218 (26.5%) of study participants have a family member(s) with NCDs. This view is echoed by a 24 years old female FGD participant as; “*… My father has been suffering from hypertension and diabetes mellitus. Once upon a time, he was admitted to the hospital due to an extremely high blood sugar level, for which I had been caring for him. And, a nurse told me that such diseases have their roots at a young age and advised me to take preventive measures such as eating vegetables, exercising regularly, avoiding high-fat foods, and getting regular health check-ups.…”*

The prevalent NCD was hypertension accounting for 187 (22.7%). This is also evidenced by IDI results. A 41 years old male public health officer stated that “*…I've been serving in this community for over 8 years, and the most common NCD I've observed is hypertension, followed by diabetes mellitus…”*

## Discussion

This study aims to determine the adequate level of knowledge of NCDs and associated factors among adult residents of the North Shewa zone, Oromia region, Ethiopia. Accordingly, the level of adequate knowledge of NCDs among residents was 33.9% (95%CI: 30.67, 37.13). Income, receiving information from health professionals, owning a TV, having a family member with NCD(s), and marital status were factors significantly associated with adequate knowledge of NCDs.

About 33.9% of the participants had an adequate level of knowledge of NCDs. The current study is consistent with a study from Rwanda (35.0%) (21). However, the prevalence of the current study is lower than the studies from Bangladesh (57.9%) (12), Spain (46.7%) (13), Saudi Arabia (43.8%) (14), and Sri Lanka (43%) (15). The variation might be due to differences in the study population, sampling techniques, and sociodemographic characteristics. In contrast, the finding of this study is higher than the study done in Malaysia (27.7%) (16). Utilization of different knowledge measuring tools may result in discrepancies. Additionally, the current magnitude is higher than a study done in China (25%) (17) and much higher than a study from Myanmar (12.5%) (18). The difference might be attributed to variation in a study population and study settings (17).

In a current study, having a family member with NCD(s) had increased the likelihood of NCDs knowledge. This is also evidenced by previous studies (17–19). Because this will lead to improved consideration of disease control for themselves eventually (17). Besides, this might be due to study subjects' involvement in caregiving to family members' with NCD(s) (18). Moreover, receiving information from health professionals increased the chance of adequate NCDs knowledge. The previous study also supports this finding (17). This is because receiving counseling services from health care providers will contribute to a discussion on various health topics, resulting in increased knowledge and understanding of NCDs.

Furthermore, having a higher income has resulted in a higher likelihood of having adequate knowledge of NCDs. This finding was supported by previous studies (12, 18). This could be explained as the presence of a concentration of risky behaviors for NCDs among the poorest residents (18). Additionally, owning a TV as a source of information is significantly associated with adequate knowledge of NCDs. The previous research also found that mass media, including television, has a massive capacity to assist healthy behavioral changes attributed to NCDs (33). Moreover, being widowed/divorced was found to have inadequate knowledge of NCDs in a current study. As a result, being widowed was associated with worse health outcomes (34, 35).

### Implications for Policy and Practice

As NCDs are one of the major health and development challenges we face today, investigating a related theme will lead to a deep understanding of the problem and searching for an effective solution like detection, screening, treatment, and palliative care. Particularly, Ethiopia's health system is working to implement awareness-raising programs on NCDs and risk factors for different segments of the population (22). Without knowledge about NCDs and their risk factors, it is difficult to achieve a reduction in the incidence and prevalence of NCDs (36). As a result, determining the level of knowledge about NCDs emphasizes the significance of conducting regular surveillance for NCDs' risk factors and initiating prevention programs (15).

### Methodological Consideration

All questionnaires were translated to the local language (Afan Oromo) by two independent bilingual translators and back-translated to English to guarantee consistency. Then it was pre-tested on 5% of the total sample size in the district which was not selected for actual study to evaluate readability, understandability, completeness, and reliability, and modification was made accordingly. Data collectors and supervisors were trained on the objectives of the study, how to collect data, and ethics. Filled questionnaires were checked for completeness and consistency on daily basis. Overall activities of the study were supervised by the supervisors and regulated by the investigators. Despite these, being a cross-sectional study, made it impossible to draw causal inferences. Additionally, a self-report technique was used to assess the knowledge, which may have resulted in recall bias and social desirability bias.

## Conclusion

This study reveals a high magnitude of inadequate level of NCDs knowledge despite its foundational ability in tackling the burden of NCDs. As a result, broadening a wider and more comprehensive health promotion strategy for the prevention of double burden of NCDs would benefit the population. Additionally, special efforts are needed both at the practice and policy levels to target disadvantaged groups, such as low-income earners, those who do not receive information from health professionals, those who do not own a television, and those who are widowed/divorced, who were found to have less knowledge of NCDs. More research is needed to elucidate the factors that can lead to poor knowledge about NCDs.

## Data Availability Statement

The original contributions presented in the study are included in the article/supplementary material, further inquiries can be directed to the corresponding author/s.

## Ethics Statement

The studies involving human participants were reviewed and approved by Salale University Institution review board. The patients/participants provided their written informed consent to participate in this study.

## Author Contributions

All authors made a significant contribution to the work reported, in the conception, study design, execution, acquisition of data, analysis and interpretation, took part in drafting, critically reviewing the article, gave final approval of the version to be published, and agree to be accountable for all aspects of the work.

## Funding

This research was funded by Salale University, Ethiopia under grant number SU/1270/21/2013.

## Conflict of Interest

The authors declare that the research was conducted in the absence of any commercial or financial relationships that could be construed as a potential conflict of interest.

## Publisher's Note

All claims expressed in this article are solely those of the authors and do not necessarily represent those of their affiliated organizations, or those of the publisher, the editors and the reviewers. Any product that may be evaluated in this article, or claim that may be made by its manufacturer, is not guaranteed or endorsed by the publisher.

## Abbreviations

CVDS: cardiovascular diseases
LMICs: low- and middle-income Countries
NCDs: non-communicable diseases
WHO: world health organization.
